# Maresin 1 Mitigates Inflammatory Response and Protects Mice from Sepsis

**DOI:** 10.1155/2016/3798465

**Published:** 2016-11-30

**Authors:** Ruidong Li, Yaxin Wang, Zhijun Ma, Muyuan Ma, Di Wang, Gengchen Xie, Yuping Yin, Peng Zhang, Kaixiong Tao

**Affiliations:** ^1^Department of General Surgery, Union Hospital, Tongji Medical College, Huazhong University of Science and Technology, No. 1277, Jiefang Ave, Wuhan 430022, China; ^2^Department of Anesthesiology and Critical Care, Union Hospital, Tongji Medical College, Huazhong University of Science and Technology, No. 1277, Jiefang Ave, Wuhan 430022, China

## Abstract

Sepsis, frequently caused by infection of bacteria, is considered as an uncontrollable systematic inflammation response syndrome (SIRS). Maresin 1 (Mar1) is a new proresolving mediator with potent anti-inflammatory effect in several animal models. However, its effect in sepsis is still not investigated. To address this question, we developed sepsis model in BALB/c mice by cecal ligation and puncture (CLP) with or without Mar1 treatment. Our data showed that Mar1 markedly improved survival rate and decreased the levels of proinflammatory cytokines in CLP mice such as interleukin-6 (IL-6), tumor necrosis factor-*α* (TNF-*α*), and interleukin-1*β* (IL-1*β*). Furthermore, Mar1 reduced serum level of lipopolysaccharide (LPS) and enhanced the bacteria clearance in mice sepsis model. Moreover, Mar1 attenuated lung injury and decreased level of alanine transaminase (ALT), aspartate transaminase (AST), creatinine (Cre), and blood urea nitrogen (BUN) in serum in mice after CLP surgery. Treatment with Mar1 inhibited activation of nuclear factor kappa B (NF-*κ*b) pathway. In conclusion, Mar1 exhibited protective effect in sepsis by reducing LPS, bacteria burden in serum, inhibiting inflammation response, and improving vital organ function. The possible mechanism is partly involved in inhibition of NF-*κ*b activation.

## 1. Introduction

Sepsis is a systematic inflammation response syndrome caused by infection of Gram-positive and/or Gram-negative microorganism and a fatal cause of human mortality [1–3]. The incidence of sepsis is high and the mortality rate reaches nearly 20 to 50% [4]. To date, although considerable progress of anti-infective therapy and organ function support technology has been made, severe sepsis and sepsis shock are still a lethal and intractable pathological condition [5]. The pathogenesis of sepsis is still not fully elucidated. Possible mechanism involves endotoxin releasing and subsequently activates immune cells to overproduce massive inflammatory mediators, followed by tissue injury and consequent multiple organ failure (MOF) syndrome. It is widely accepted that the initial stage of this signaling pathway involves toll-like receptors (TLRs) activated by certain bacterial component like lipopolysaccharide (LPS). Then, nuclear factor kappa B (NF-*κ*b) system is activated and regulates transcription of inflammation gene which controls expression of various cytokines and inflammatory mediators [2]. Excessive inflammation response and subsequent immune disorder cause multiple organs dysfunction and high incidence of mortality.

Maresin 1 is a new proresolving mediator which is derived from docosahexaenoic acid (DHA) [6]. Recently, it was demonstrated in vivo and in vitro that maresin 1 can enhance inflammation resolution and show protective properties by inhibiting inflammatory response [7, 8]. During septic shock, many inflammatory mediators, such as interleukin-1*β*, tumor necrosis factor-*α*, and interleukin-6, increased dramatically and caused tissue or organ damage [9]. Severe sepsis is frequently accompanied with acute respiratory distress syndrome (ARDS), hepatic dysfunction, and other organ failures, which lead to poor prognosis [10]. Many previous studies have demonstrated that proresolving mediators, like maresin 1, had protective effect in several organ specified injury models, such as acute lung injury, colitis, liver injury, and lung fibrosis [7, 11–14]. Cecal ligation and puncture (CLP) is an experimental model of lethal sepsis, which can simulate the whole pathology of sepsis which occurs in clinic patients [2]. To date, the protective effects of maresin 1 have not been investigated in a murine model of CLP induced sepsis. In this context, we designed the present experiment with hypothesis that maresin 1 has potential therapeutic effect in a murine CLP sepsis model.

## 2. Material and Method

### 2.1. Animals

All BALB/c mice were purchased from the animal experimental center of Wuhan University, aged 9 to 10 weeks and weighted range from 22 to 25 g. All animals were maintained in a pathogen-free room with standard laboratory diet and water ad libitum and the room temperature was kept between 22 to 24°C with 12 h light-dark cycles. All experimental procedures involving mice were approved by the Animal Care and Use Committee of Tongji Medical College of Huazhong University of Science and Technology.

### 2.2. Regents

7R-maresin 1 was bought from Cayman Chemical (Ann Arbor, MI, USA). Alanine transaminase (ALT), aspartate transaminase (AST), creatinine (Cre), and blood urea nitrogen (BUN) detection kits were purchased from Nanjing Jiancheng Institute of Biotechnology (Nanjing, China). Mouse tumor necrosis factors-*α* (TNF-*α*), interleukin-1*β* (IL-1*β*), and interleukin-6 (IL-6) enzyme-linked immunosorbent assay (ELISA) kits were purchased from Dakewe Bioengineering Co., Ltd. (Shenzhen, China). NF-*κ*b p65 antibody and *β*-actin were obtained from Santa Cruz Biotechnology. Lamin B1 antibody was purchased from Epitomics (Burlingame, CA).

### 2.3. Experimental Procedure and Treatments

The CLP was performed as described before with minor alteration. Briefly, mice were anesthetized by pentobarbital sodium (0.25%, 50 mg/kg); then an abdominal middle incision was made to expose cecum. The cecum was isolated and ligated at midpiece of the cecum with 4-0 silk suture. Then, the cecum was punctured with 18-gauge needle. The cecum was placed back to peritoneal cavity and the incision was closed in layers. After that, 1 mL sterile saline was injected subcutaneously for fluid resuscitation. Sham group was treated with same procedure as CLP group without cecum puncture and ligation. To explore protective effects of maresin 1, mice were divided into four groups: (1) sham+Mar1 group: mice underwent sham operation followed by Mar1 administration; (2) sham group: mice underwent sham operation followed by vehicle administration; (3) CLP group: mice were given CPL followed by administration of vehicle (0.1 mL sterilized saline); (4) maresin 1 group: mice were subjected to CLP operation followed by treatment with maresin 1. 1 ng. Mar1 (Cayman Chemical, Ann Arbor, MI, USA) was diluted in 0.1 mL saline and then was injected via the tail vein. The dose of Mar1 was chosen from what we reported before [7]. 24 h after operation, blood sample was collected from right ventricle with heparinized syringe and peritoneal lavage was obtained by injecting 1.5 mL sterile saline into peritoneal cavity. The lung sample was acquired at the same time.

### 2.4. Survival Analysis

The CLP treated mice were randomized to receive tail vein injection of maresin 1 or vehicle. And survival was recorded every 24 h for a total of 7 days.

### 2.5. Determination of LPS Serum Level in CLP Mice

After 12 hours of CLP treatment, the mice were sacrificed by administrating overdose of sodium pentobarbital. The blood samples were obtained from left ventricle. To separate serum from blood, the samples were centrifuged for 3000 g for 18 min. A Limulus Amebocyte Lysate (LAL) test was used to measure serum LPS level using ToxinSensor™ Chromogenic LAL Endotoxin Assay Kit (GenScript, Nanjing, China) according to manufacturer's instructions.

### 2.6. Bacterial Culture

To measure quantity of bacteria, peritoneal lavage or blood was diluted with sterile saline at 1 : 10 to 1 : 10^6^. Each dilution was cultured on a tryptic soy blood agar plate in an aerobic conditions for 24 h at 37°C. Then, colony-forming units (CFU)/mL were calculated.

### 2.7. Histopathology Analysis

The lung specimens were fixed in 4% paraformaldehyde for 24 h, embedded in paraffin, cut into sections, and stained with hematoxylin and eosin. The pathological alteration was recorded using light microscope (Olympus IX71, Tokyo, Japan) by pathologist who was blind to experimental groups. The lung injury scores were calculated according to a recent reported criterion [12]. Briefly, an overall score of between 0 and 1 was used to evaluate degree of lung injury. The injury score was calculated as the following formula: lung injury score = (20 × *A* + 14 × *B* + 7 × *C* + 7 × *D* + 2 × *D*)/(number of fields × 100). The parameters *A*, *B*, *C*, *D*, and *E* were described in Table 1.

### 2.8. Cytokine Assays

Levels of IL-6, TNF-*α*, and IL-1*β* in serum were measured by respective murine ELISA kit (Dakewe Bioengineering Co., Ltd., Shenzhen, China), according to manufacturer's instructions.

### 2.9. Determination of Liver and Kidney Function

Serum was obtained by centrifugation of blood sample at 1500 g for 15 min. Then, ALT, AST, Cre, and BUN were measured by respective kits (Nanjing Jiancheng Institute of Biotechnology, Nanjing, China) according to the manufacturer's instruction.

### 2.10. Western Blot

Blood sample were collected from mice 12 hours after CLP operation as described previously. The peripheral blood mononuclear cells (PBMC) were collected using Ficoll lymphocyte separation medium (TBD Science, Tianjin, China). Total protein of PBMC was extracted using Radio Immunoprecipitation Assay kits (Beyotime Biotechnology Company, Jiangsu, China) according to the manufacturer's instruction. Cytoplasmic and nuclear protein were extracted with NE-PER Nuclearand Cytoplasmic Extraction Reagents (Pierce Biotechnology) according to the manufacturer's instruction. The protein concentrations were determined by BCA protein assay kit (Beyotime Biotechnology Company, Jiangsu, China). Then protein extracts were fractionated in 10% polyacrylamide sodium dodecyl sulfate gels and transferred to polyvinylidene fluoride (PVDF) membrane. The membrane was blocked with 5% fat-free milk in 0.1% Tween 20 tris-buffered saline (TBST) buffer for 2 hours and then was incubated with antibody against *β*-actin (1 : 1000), NF-*κ*b (1 : 1000), and lamin B1 (1 : 1000) overnight at 4°C. Then, the membrane was incubated with horseradish peroxidase (HRP) conjugated secondary antibody for 1 hour. An ECL chemiluminescence system was used to visualize the binding and images were developed using UVP imaging system.

### 2.11. Statistical Analysis

All data were expressed as means ± SEM. Survival rate was analyzed using the log-rank test. All comparisons among groups were performed by one-way analysis of variance (ANOVA) and intergroup comparison is applied with Student-Newman-Keuls post hoc analysis. A value of *P* < 0.05 was considered to be statistically significant for all calculations. All statistical analyses were performed using SPSS software version 17.0 (SPSS Inc., Chicago, IL).

## 3. Results

### 3.1. Maresin 1 Improved the Survival Rate and Weight Loss in Sepsis Mice

**Figure 1:**
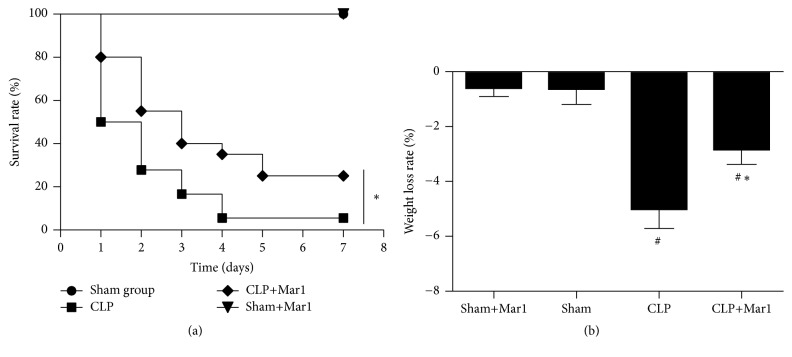
Improvement of survival rate (*n* = 20) and weight loss by Mar 1 in CLP mice. (a) Survival rate was significantly improved by Mar 1 administration. (b) Weight loss decreased with treatment of Mar 1. Survival rate was analyzed using the log-rank test. ^#^ *P* < 0.05 versus the sham group. ^*∗*^ *P* < 0.05 versus the CLP group.

To explore whether maresin 1 can improve CLP mice, survival was recorded for 7 days. As shown in Figure 1(a), there was no difference of survival rate between sham group and sham+Mar1 group. However, CLP significantly elevated the mortality rate in mice. Significantly, treatment of Mar1 can improve the survival rate in CLP mice, which indicated protective effect of Mar1 in sepsis. In Figure 1(b), the body weight significantly decreased after CLP. Interestingly, the Mar1+CLP group showed lower rate of weight loss compared to CLP group. Weight loss showed no difference between sham and sham+Mar1 group.

### 3.2. Maresin 1 Reduced Serum Level of LPS

**Figure 2:**
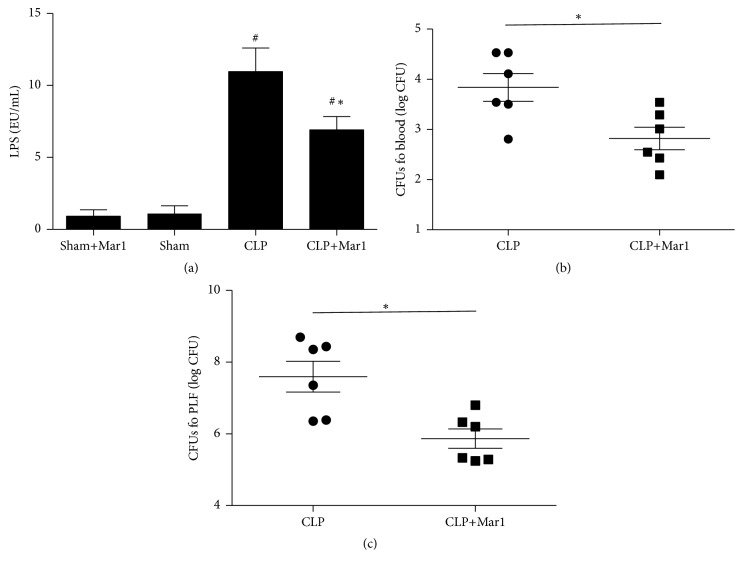
Mar1 decreased LPS and bacterial burden in CLP mice. (a) The level of serum LPS in CLP mice. The CFUs (colony-forming units) in blood (b) and in peritoneal lavage (c) in CLP mice. Data are expressed as means ± SEM. *n* = 6. ^#^ *P* < 0.05 versus the sham group. ^*∗*^ *P* < 0.05 versus the CLP group.

To explore whether maresin 1 can reduce LPS level in CLP mice, the serum LPS was evaluated. In present study, LPS level significantly increased after CLP surgery compared to sham and sham+Mar1 group (Figure 2(a)). Meanwhile, the serum level of LPS markedly decreased with maresin 1 treatment.

### 3.3. Maresin 1 Reduced Bacterial Burden in Sepsis Mice

To explore whether maresin 1 can enhance bacterial clearance in CLP mice, the CFU in blood and lavage fluid was measured. We found that bacterial colony formation in blood and lavage fluid markedly decreased in Mar1+CLP group compared with CLP group (Figures 2(b) and 2(c)). The data indicated that maresin 1 promoted bacterial clearance in blood and lavage fluid in sepsis mice.

### 3.4. Maresin 1 Inhibited CLP Induced Proinflammatory Cytokines Activities

**Figure 3:**
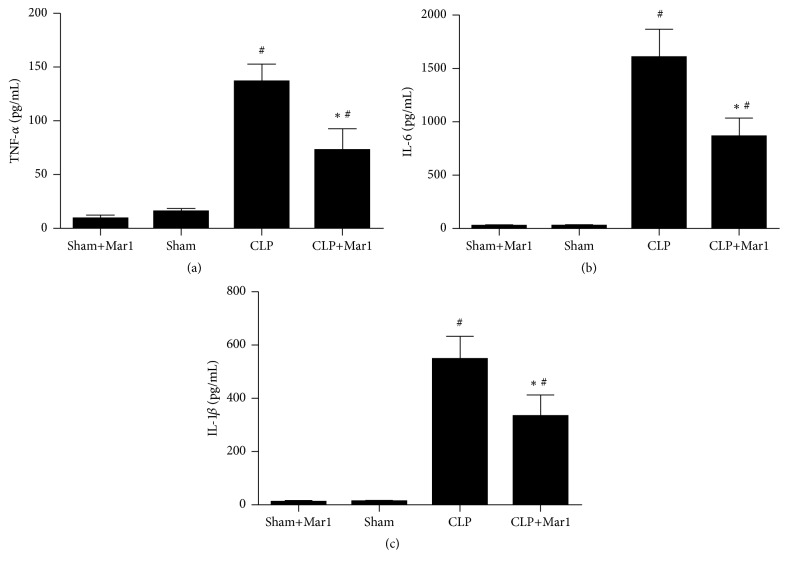
Inhibition of proinflammatory cytokines by Mar1 in CLP animals. Levels of TNF-*α* (a), IL-6 (b), and IL-1*β* (c) in serum were determined by enzyme-linked immunosorbent assay. Data are expressed as means ± SEM. *n* = 6. ^#^ *P* < 0.05 versus the sham group. ^*∗*^ *P* < 0.05 versus the CLP group.

Overwhelming proinflammatory cytokines play an important role in pathologic process of sepsis. In our study, we found that sham operation did not increase inflammatory cytokines. However, levels of proinflammatory cytokines such as IL-6, TNF-*α*, and IL-1*β* significantly increased after CLP operation compared to sham and sham+Mar1 group. Significantly, the activity of IL-6, TNF-*α*, and IL-1*β* in CLP mice was inhibited after treatment with maresin 1 (Figure 3).

### 3.5. Maresin 1 Alleviated Lung Histopathologic Change in CLP Mice

**Figure 4:**
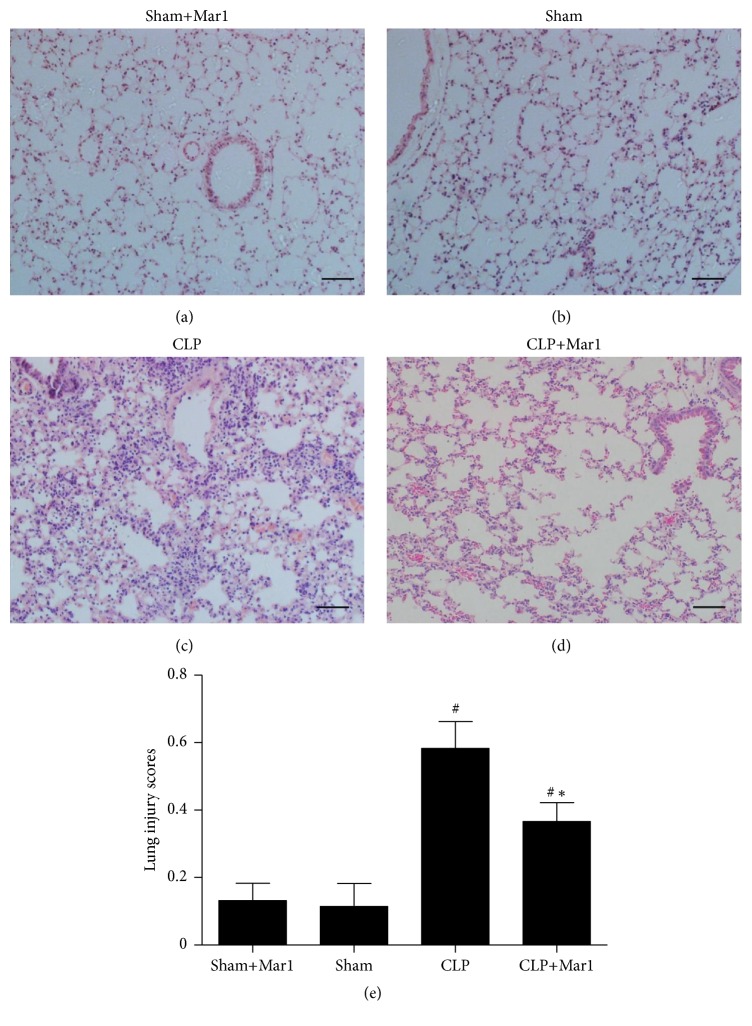
Improvement of lung histopathological changes by Mar1 in CLP mice. Lung tissues were obtained 24 hours after CLP. (a, b, c, and d) Representative micrographs from sham+Mar1, sham, CLP, and CLP+Mar1 group are shown. (e) Lung injury score in each group. Data are expressed as means ± SEM. Black bars represent 100 *μ*m. *n* = 5. ^#^ *P* < 0.05 versus the sham group. ^*∗*^ *P* < 0.05 versus the CLP group.

Lung injuries are frequently involved in pathologic process of sepsis. Sham+Mar1 and sham mice treated with vehicle showed normal lung architecture. As shown in Figure 4. The lung tissue in CLP group displayed significant interstitial edema and leukocyte infiltration (Figure 4(c)). In contrast, administration of maresin 1 after CLP markedly diminished those histopathologic changes (Figure 4(d)). Accordingly, the lung injury scores (lung injury score: sham+Mar1 = 0.148 ± 0.039, sham = 0.136 ± 0.064, CLP = 0.592 ± 0.080, and CLP+Mar1 = 0.370 ± 0.055) were in accordance with the histopathologic alteration in lung tissues (Figure 4(e)).

### 3.6. Maresin 1 Improved Liver and Kidney Function after CLP Surgery

**Figure 5:**
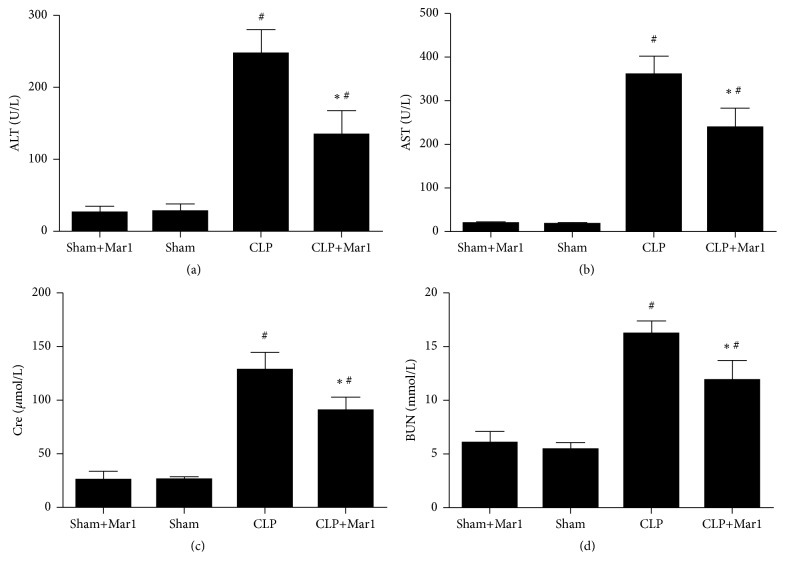
Improvement of liver and kidney functions of mice by Mar1 after CLP surgery. The change of serum ALT (a) and AST (b) activities by Mar1 in CLP mice. The change of Cre (c) and BUN (d) in serum by Mar1 in CLP animals. *n* = 6. Data are expressed as means ± SEM. ^#^ *P* < 0.05 versus the sham group. ^*∗*^ *P* < 0.05 versus the CLP group.

Liver and kidney dysfunction frequently occur during the pathologic process of severe sepsis. In sham and Mar1+sham group, AST, ALT, Cre, and BUN stayed at normal level. The levels of AST, ALT, Cre, and BUN markedly increased after CLP surgery. Nevertheless, treatment of maresin 1 significantly decreased AST, ALT, Cre, and BUN level in serum (Figure 5).

### 3.7. Maresin 1 Inhibited NF-*κ*b p65 Nuclear Translocation in PBMC

**Figure 6:**
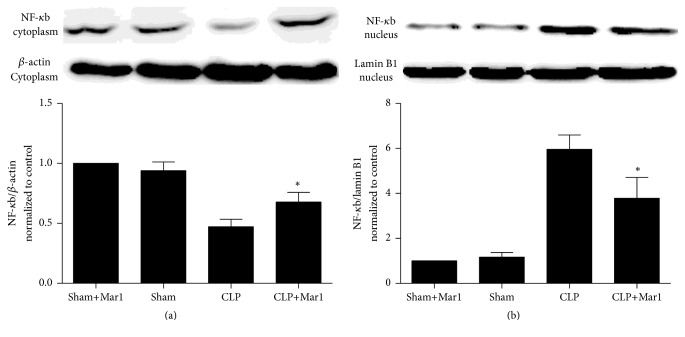
Translocation of PBMC NF-*κ*B p65 subunit was inhibited by Mar1 in CLP mice. (a) NF-*κ*B p65 in cytoplasm decreased after CLP surgery but increased by Mar1. (b) NF-*κ*B p65 increased in CLP mice, but Mar1 decreased level of NF-*κ*B p65 in nucleus. NF-*κ*B p65 band from the experiments was normalized by *β*-actin or lamin B1. *n* = 6. Data are expressed as means ± SEM. ^*∗*^ *P* < 0.05 versus the CLP group.

The activation of NF-*κ*b pathway plays a pivotal role in inflammation. As shown in Figure 6, the level of NF-*κ*b p65 in cytoplasm and nucleus showed no difference between sham and Mar1+sham group, which indicated that sham operation did not activate NF-*κ*b p65 pathway. However, NF-*κ*b p65 level in cytoplasm decreased significantly in CLP group, while NF-*κ*b p65 level in nucleus increased markedly, which indicated that NF-*κ*b p65 translocation from cytoplasm to nucleus was enhanced after CLP treatment compared to sham and Mar1+sham group. In contrast, this translocation was inhibited with maresin 1 administration. Therefore, activation of NF-*κ*b pathway was inhibited with treatment of Mar1.

## 4. Discussion

Sepsis is considered as uncontrollable inflammatory reaction in severe infection. The murine CLP model is widely accepted as a sepsis model in current sepsis research. Previous reports indicated that maresin 1 greatly mitigated endotoxin induced lung injury [7]. As endotoxin is also main cause of sepsis, this study was designed to determine the effects of maresin 1 on sepsis.

In present study, the sepsis model was successfully elicited. Our finding first time suggested that maresin 1 improved the survival rate in sepsis mice induced by CLP and indicated certain beneficial effects, including decreasing bacterial burden and mitigating excessive inflammatory reaction. Also, our work indicated that, at least in part, the possible mechanism is involved in organ functional protection in sepsis. The occurrence of sepsis and consequent MOF is highly associated with bacteria and endotoxin translocation, which can trigger excessive inflammatory response and organ dysfunction [15]. LPS is the main components of the cell wall of Gram-negative bacteria and many studies proved that LPS could induce lethal inflammatory response, causing MOF and even death [9, 16]. Correspondingly, removal of LPS in animals was effective in treatment of sepsis [16]. In our present study, we found that LPS level in serum was significantly reduced by maresin 1. Apart from that, the bacterial clearance in blood and peritoneal lavage was promoted. Therefore, these suggested maresin 1 might have protective effect in sepsis through reducing source of endotoxin.

Additionally, uncontrolled inflammatory response is another pathologic hallmark of sepsis [17]. LPS can promote overexpression of proinflammatory cytokines including IL-6, IL-1*β*, and TNF-*α*, which was involved in subsequent sepsis [18, 19]. It was previously found that TNF-*α* played a pivotal in sepsis which could initiate systematic inflammatory response [9]. IL-6 is also associated with CLP induced sepsis, and it was reported that selective blockade of IL-6 transsignaling improves survival rate [20]. In addition, IL-6, IL-1*β*, and TNF-*α* had a central role in activating cytokine cascade response in sepsis and partly involved in pathogenesis of MOF [21]. Therefore, decreasing the activity of those proinflammation cytokines might have potential protective effect and avoid excessive inflammation reaction during sepsis. In our study, the serum proinflammatory cytokines such as IL-6, IL-1*β*, and TNF-*α* were greatly inhibited by maresin 1 in CLP mice. All these suggested anti-inflammation effect of maresin 1 in CLP sepsis model.

Lung, liver, and kidney are important organs which are susceptible to be attacked during sepsis [22]. Their poor function and state are frequently related to poor prognosis. Activity of AST and ALT is widely used to evaluate hepatic function, and kidney function is frequently assessed using Cre and BUN in serum. In our studies, pathohistologic changes of lung tissues in CLP mice were significantly improved with treatment of maresin 1. Combined with survival rate improvement in maresin 1 treated CLP mice, all these indicated that maresin 1 had potential protective effect by ameliorating function of important organs during sepsis.

Furthermore, we investigated possible mechanism and signaling pathway involved in this protective effect in sepsis. PBMC is one of main inflammatory mediators' sources during sepsis process. NF-*κ*b pathway is considered as a key regulator of inflammatory gene expression [23, 24]. It was reported that NF-*κ*B Inhibition mitigated lung injury induced by CLP [25]. To the best of our knowledge, the activation of NF-*κ*b is related to phosphorylation. Normally, various stimulus such as growth factors, cytokines, and stress can activate NF-*κ*b pathway. This results in translocation of NF-*κ*b into nucleus and activation of transcription of certain gene. Previous reports have identified that LPS could enhance translocation of NF-*κ*b p65 from the cytoplasm to the nucleus [24, 26]. Thus, the transcription of inflammatory cytokines was activated. And some reports indicated that NF-*κ*b pathway was activated during sepsis and increased expression of proinflammatory cytokines such as IL-6, IL-1*β*, and TNF-*α*, leading to excessive inflammation response [23, 27]. In our present study, the activation of NF-*κ*b p65 in PBMC was markedly inhibited with maresin 1 treatment in CLP animals. These data suggested that the protective effect of maresin 1 in sepsis might be associated with inhibition of activation of NF-*κ*b pathway.

## 5. Conclusion

Our results suggested maresin 1 markedly protected mice from CLP induced sepsis by reducing systematic inflammation response. The possible mechanism, at least in part, might be associated with its clearance of bacterial burden and consequent less LPS in mice, subsequently suppressing LPS induced NF-*κ*b p65 pathway, downregulation of inflammatory response, and protection of functions of important organs. Taken together, maresin 1 can be a potential therapeutic agent for treatment of sepsis in the future.

## Figures and Tables

**Table 1:** Lung injury score parameter.

Parameter	Score		
	0	1	2
*A*: neutrophils in the alveolar space	0	1–5	>5
*B*: neutrophils in the interstitial space	0	1–5	>5
*C*: hyaline membranes	0	1	>1
*D*: proteinaceous debris filling the airspaces	0	1	>1
*E*: alveolar septal thickening	<2*x*	2*x*–4*x*	>4*x*
